# Treatment of restless legs syndrome/Willis-Ekbom disease with selenium

**Published:** 2016-10-07

**Authors:** Jan Ulfberg, Romana Stehlik, Ulrike Mitchell

**Affiliations:** 1 Sleep Center, Capio Health Center, Hamnplan, Örebro, Sweden; 2 Pain Center, Uppsala University Hospital, Uppsala, Sweden; 3 Department of Exercise Sciences, Brigham Young University, Provo, United States

**Keywords:** Restless Leg Syndrome, Selenium, Minerals, Pramipexole

Restless legs syndrome/Willis-Ekbom disease (RLS/WED) is a chronic neurosensory disorder that is characterized by a strong urge to move, accompanied by an uncomfortable paresthesia in the legs which affects about 5-15 percent of adult people in industrialized countries.^1^ RLS/WED is a puzzling disease, mostly because of the uncertainty of its etiology. There seems to be a genetic component, but it is also known that RLS/WED may be caused by other underlying pathologies and is treated by remedying those conditions. Another highly researched area in the quest to find an etiology and treatment for RLS/WED is the brain dopamine system; in fact, dopaminergic medication is considered the first line of treatment for RLS/WED that is not caused by other underlying factors.^1^

There is growing evidence, suggesting that selenium plays an important role in many body functions, and seems to be an important regulator of brain function as well.^2^ Selenium has a strong anti-oxidant action.^2^ The body concentration of selenium is related to soil concentration, which is low in Sweden. 

We report the successful treatment of 3 patients with severe RLS/WED with the intake of selenium for 6 months; 

Three female patients, aged 25-60 years, were all suffering from severe to very severe RLS/WED since childhood. Severity was measured by using the International Restless Legs Scale (IRLS), a 10-item questionnaire developed by the International Restless Legs Syndrome Study Group (IRLSSG).^1^ Each question has five response options, ranging from “0” to “4”. Hence, the total score can range from “0” to “40”, indicating “no symptoms” to “very severe symptoms”, respectively. The subjects’ IRLS scores were between 25 and 38. The subject with the IRLS score of 25 was being treated with the dopaminergic drug pramipexole, 0.18 mg in the evening. No one was a smoker, and they were healthy and without any other medical treatment or alternative medicine. Height, weight, blood pressure, hemoglobin, the vitamins folic acid and B12, kidney functions and iron status were within normal limits. 

All 3 patients started to take selenium yeast 100 micrograms daily. This was bought over-the-counter. No other instructions were given. 

Six months later, the patients presented at the clinic and were re-assessed. Their RLS/WED symptoms were substantially reduced to “moderate”, represented by their IRLS scores of 10 to 18. The patient who was taking pramipexole had discontinued the intake of the drug. All the patients reported independently from each other that they did not experience any changes initially, but that, after 4 months of treatment, there was a slow and steady reduction of their RLS-related symptoms. None of the patients reported any side effects. The fact, that the patients in this report noticed reduction of their symptoms first after 4 months of treatment would indicate that the placebo efficacy here is lower than expected. Serum-selenium was not recorded among these three patients, but is known to be lower than recommended in this part of the world. 

The literature on selenium in RLS/WED is minimal. However, the first and only report in this context, a placebo-controlled trial by Rahimdel et al., showed the RLS/WED symptom-relieving benefits of selenium salt, taken orally, 50 or 200 micrograms per day. The positive outcome was recorded after only one month of treatment.^3^

Interesting in this context is that serum-selenium is low in pregnant women and in patients with end-stage renal disease (ESRD) on hemodialysis,^4^^,^^5^ two groups of patients known to have with a very high prevalence of RLS/WED.^1^ However, there are no data on selenium-status in pregnant women, in patients with ESRD or with other pathologies, who are also suffering from RLS/WED; so, further studies in this context are warranted.

It is possible, at least in theory, that selenium can reduce the symptoms of RLS/WED as it has been proposed that selenium may work on the function of the dopaminergic system.^2^ As well, it is known that patients with RLS/WED are under oxidative stress.^1^ Thus, given the fact, that selenium is a potent antioxidant, its mechanism of action could be related to its ability to neutralize the reactive intermediates. Another possible working mechanism could be through the positive effect it has on endothelial function. 

In order to explore the efficacy of selenium in RLS/WED, future randomized clinical trials would be of great interest and value. Multiple side effects of the dopaminergic drugs, such as emerging problems of augmentation, motivate further research on alternative treatments of this common ailment. If selenium has an efficacy in reducing the symptoms of RLS/WED, future studies might also explore if selenium just compensates for a selenium deficiency in the body or if it has indeed a drug effect.

## Notes:

**How to cite this article:** Ulfberg J, Stehlik R, Mitchell U. Treatment of restless legs syndrome/Willis-Ekbom disease with selenium. Iran J Neurol 2016; 15(4): 235-6.
